# Supplementary material for: Unconventional binding of calmodulin to CHK2 kinase inhibits catalytic activity
Source: Biochem J. 2025 Dec 3;482(23):1759–77. doi: 10.1042/BCJ20253431 (PMC12751073; doi:10.1042/BCJ20253431)

SUPPLEMENTARY INFORMATION

Unconventional binding of Calmodulin to CHK2 kinase inhibits catalytic activity

Christopher R. Horne[1,2,3*], Tingting Wang[4], Samuel N. Young[1], Toby A. Dite[1,2], Hunter G. Nyvall[5], Sushant Suresh[5], Katherine A. Davies[1,2], Abner Gonzalez Castro[1,2], Vineet Vaibhav[1,2], Lucy J. Mather[1], Laura F. Dagley[1,2], Matthew J. Belousoff[3], Gerard Manning[6], Anthony R. Means[7], John E. Burke[5,8], Janni Petersen[4], John W. Scott[3,9,10,11*], James M. Murphy[1,2,3,11*]

[1]Walter and Eliza Hall Institute of Medical Research, 1G Royal Parade, Parkville, Victoria 3052, Australia

[2]Department of Medical Biology, University of Melbourne, Parkville, Victoria 3052, Australia

[3]Drug Discovery Biology, Monash Institute of Pharmaceutical Sciences, Monash University, Victoria 3052, Australia

[4]College of Medicine and Public Health, Flinders University, Adelaide, South Australia 5001, Australia

[5]Department of Biochemistry and Microbiology, University of Victoria, Victoria, BC, Canada

[6]NuaBio Research, Burlingame, CA 94010, USA

[7]Molecular and Cellular Biology, Baylor College of Medicine, Houston, TX, 77030, USA

[8]Department of Biochemistry and Molecular Biology, University of British Columbia, Vancouver, BC, Canada

[9]The Florey Institute of Neuroscience and Mental Health, Parkville, Victoria 3052, Australia.

[10]St Vincent's Institute of Medical Research, Fitzroy, Victoria 3065, Australia.

[11]These authors contributed equally to the supervision of the study

[*]Please direct correspondence to horne.c@wehi.edu.au (CRH), john.scott@monash.edu (JWS) or jamesm@wehi.edu.au (JMM)

SUPPLEMENTARY TABLES

Supplementary Table 1. Oligonucleotide sequences

CHK2 2 IF fwd	5'-GTATTTTCAGGGATCCTCTCGGGAGTCGGATGTTGAG-3'
CHK2 553 STOP IF rev	5'-CAGTCACGATGAATTCACAACACAGCAGCACACACAG-3'
CHK2 210 IF fwd	5'-GTATTTTCAGGGATCCTCAGTTTATCCTAAGGCATTAAGAG-3'
CHK2 531 STOP IF rev	5'-CAGTCACGATGAATTCACTCGGCACCCTCGGCTTC-3'
pcDNA XhoI N-FLAG‡	5'-cgcCTCGAGATGGATTACAAGGATGACG-3'
pcDNA STOP HindIII rev	5'-cgcAAGCTTTCACAACACAGCAGCAC-3'
pFB CHK2 IF fwd	5'-ACCATGTCGTACTACatgtcccctatactaggttattggaaaattaagg-3'
pFB CHK2 IF rev	5'-GATGTCGTTCAGACCTCACAACACAGCAGCACACACA-3'
pFB vector fwd	5'-GGTCTGAACGACATCTTCGAGG-3'
pFB vector rev	5'-GTAGTACGACATGGTTTCGGACCG-3'
hCaM BamHI 2 fwd	5'-cgcGGATCCgctgatcagctgaccgaagaacag-3'
hCaM 149 STOP NotI rev	5'-cgcGCGGCCGCTCattttgcagtcatcatctgtacgaattc-3'
CHK2 K373 sense	5'-aaacTATGTGGAACCCCCACCTAC-3'
CHK2 K373 antisense	5'-caccGTAGGTGGGGGTTCCACATA-3'
CHK2 CRISPR screen fwd	5′-GAGACACTGGGGTCTAAGAACCATGTAG-3′
CHK2 CRISPR screen rev	5′-GGTGGTGTGCATCTGTAGTCCCAG-3′
CRISPR sequence fwd	5′-GAGACACTGGGGTCTAAGAACCATGTAG-3′

‡ Restriction sites underlined

Supplementary Table 2. HDX-MS data analysis statistics

	CHK2 apo	CHK2 + CaM (15μM)
HDX reaction details	%D_2O = 55.5%<br>$pH_{(read)}$ = 7.5<br>Temperature = 20 °C	%D_2O = 55.5%<br>$pH_{(read)}$ = 7.5<br>Temperature = 20 °C
HDX time course	3s at 0C, 3s, 30s, 300s, 3000s at 18C	3s at 0C, 3s, 30s, 300s, 3000s at 18C
HDX controls	N/A	N/A
Back-exchange	Corrected based off %D_2O	Corrected based off %D_2O
Number of unique peptides	85	85
Sequence coverage	77.4%	77.4%
Average peptide length	14.1	14.1
Average peptide redundancy	2.2	2.2
Replicates	3	3
Repeatability	Average StDev = 0.8%	Average StDev = 0.8%
Significant differences in HDX	>5% and >0.4 Da and unpaired t-test <0.01	>5% and >0.4 Da and unpaired t-test <0.01

SUPPLEMENTARY FIGURES

Supplementary Figure 1 | Orthogonal activity and CHK2 phosphorylation status. (A) In vitro kinase activities of immunoprecipitated full-length CHK2 and recombinant CHK2 kinase domain (amino acids 210-531), measured by ADP-Glo assay, in the presence (red) or absence (white) of Ca^{2+}-CaM. Individual data points are plotted; the bar and error bars shown represent mean ± SD of three independent assays. Statistical analysis was performed by two-way ANOVA; **** signifies $P<0.0001$. **(B)** Phos-tag gel analysis of immunoprecipitated full-length CHK2 wild-type treated with λ phosphatase prior to radiometric assay. Gel was transferred to PVDF membrane, blocked and probed by anti-FLAG. **(C)** Radiometric assay of immunoprecipitated full-length CHK2, in the presence or absence of Ca^{2+}-CaM, with (blue) or without (white) λ phosphatase treatment. Statistical analysis was performed by two-way ANOVA; **** signifies $P<0.0001$; n.s = non-significant. **(D)** Intact mass spectrometry analysis of recombinant CHK2 kinase domain treated with λ phosphatase prior to radiometric assay. **(E)** Radiometric assay of recombinant CHK2 kinase domain, in the presence or absence of Ca^{2+}-CaM, with (blue) or without (white) λ phosphatase treatment. Statistical analysis was performed by two-way ANOVA; **** signifies $P<0.0001$; n.s = non-significant.

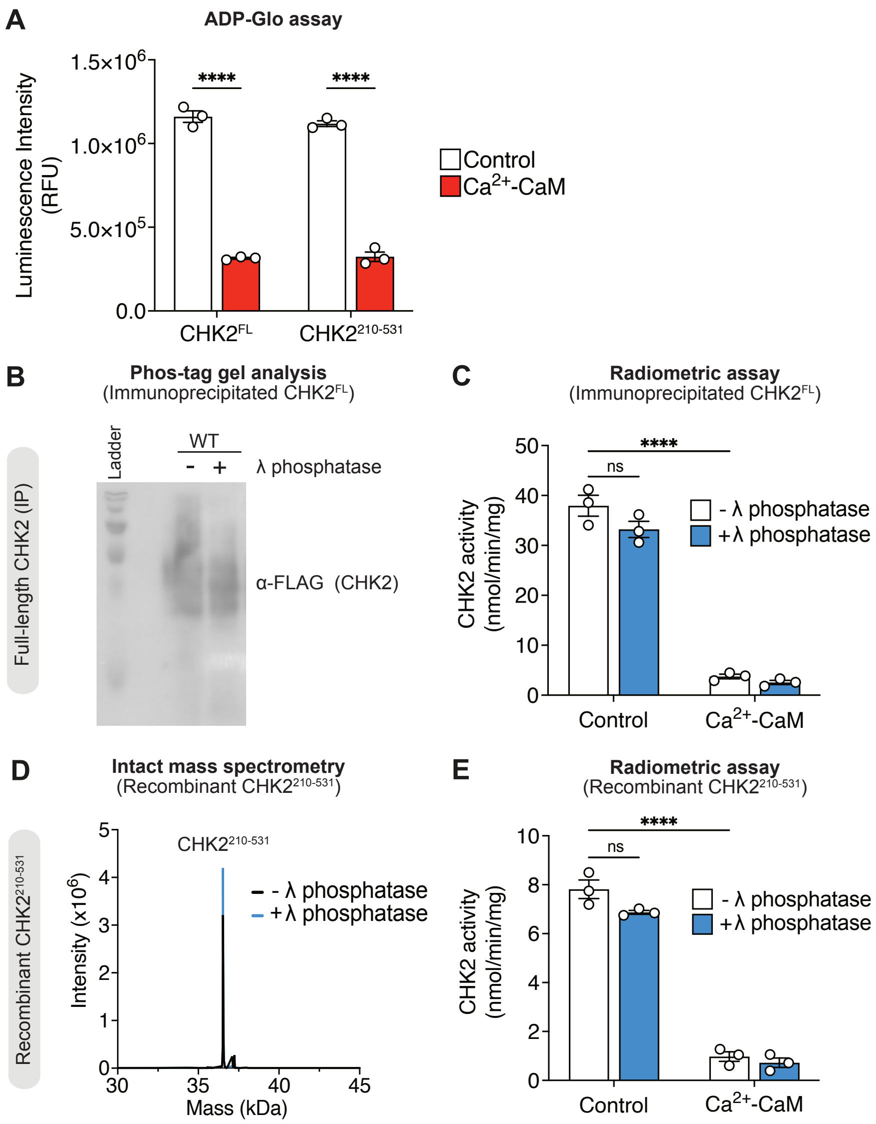
A
ADP-Glo assay
Luminescence Intensity (RFU)
1.5x10^6
1.0x10^6
5.0x10^5
0.0
****
****
CHK2^FL
CHK2^210-531
Control
Ca2+-CaM
B
Phos-tag gel analysis
(Immunoprecipitated CHK2^FL)
Full-length CHK2 (IP)
Ladder
WT
- +
λ phosphatase
α-FLAG (CHK2)
C
Radiometric assay
(Immunoprecipitated CHK2^FL)
CHK2 activity (nmol/min/mg)
50
40
30
20
10
0
****
ns
- λ phosphatase
+λ phosphatase
Control
Ca2+-CaM
D
Intact mass spectrometry
(Recombinant CHK2^210-531)
Recombinant CHK2^210-531
CHK2^210-531
Intensity (x10^6)
5
4
3
2
1
0
- λ phosphatase
+λ phosphatase
30
35
40
45
Mass (kDa)
E
Radiometric assay
(Recombinant CHK2^210-531)
CHK2 activity (nmol/min/mg)
10
8
6
4
2
0
****
ns
- λ phosphatase
+λ phosphatase
Control
Ca2+-CaM

Supplementary Figure 2 | Direct interaction between CHK2 and Ca^{2+}-CaM. (A) Double referenced SPR sensorgram for titration of CaM against immobilised CHK2. Independent repeats are shown (other shown in Figure 1D), where the sensorgram is color-coded based on concentration. **(B)** Steady state analysis is shown (blue), along with the dissociation constant.

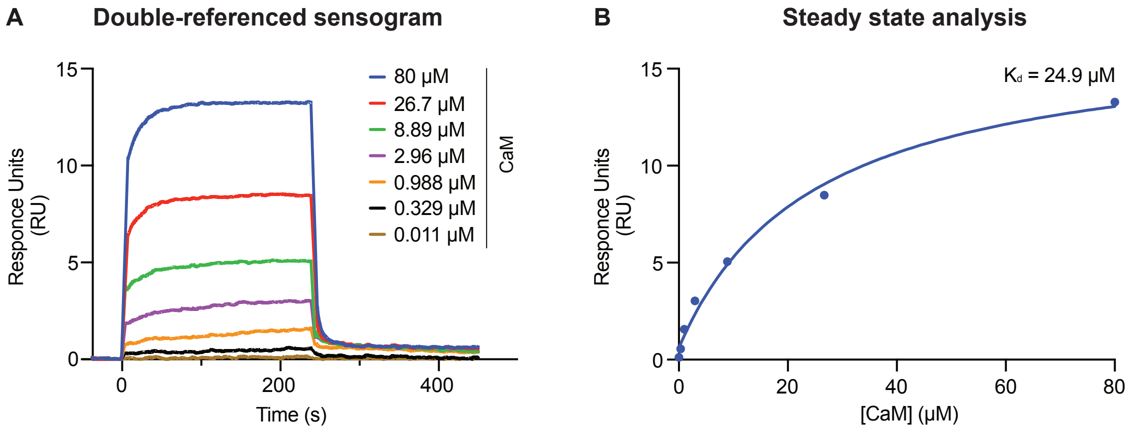

Supplementary Figure 3 | Chemical crosslinking gel analysis. (A) SDS-PAGE analysis of recombinant full-length CHK2 alone and with an increasing concentration of the zero-length crosslinker, DMTMM. **(B)** SDS-PAGE analysis of recombinant full-length CHK2 in the presence and absence of the photoactivatable crosslinker, SDA. Molecular weight markers are shown. Signal for CHK2, Calmodulin (CaM) and CHK2:CaM complex is annotated.

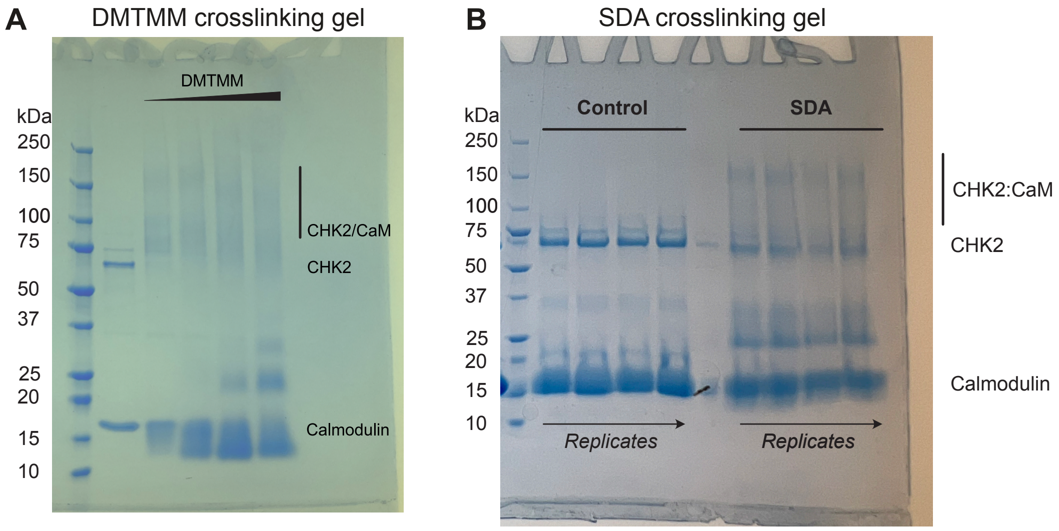

Supplementary Figure 4 | Purification, activity and stability of recombinant CHK2 constructs. (A) Reducing SDS-PAGE analysis of the purified CHK2 full-length and kinase domain (amino acids 210-531) constructs visualized by stain-free imaging. Positions of molecular weight standards (first lane) are annotated on the left of the gel. The purity of the shown protein preparations are representative of at least 3 independent expressions and purifications for each construct. **(B)** Radiometric assay of recombinant wild-type CHK2 kinase domain and DMTMM cross-linked lysine mutants in the presence (red) or absence (white) of 200 µM $CaCl_2$, 1 mM CaM for 10 min. Data represent mean ± SD; n = 3. Statistical analysis was performed by two-way ANOVA; * and **** signifies P<0.1, and P<0.0001, respectively; n.s = non-significant. **(C)** Thermal shift curves of purified CHK2 full length and kinase domain (amino acids 210-531) constructs, performed using differential scanning fluorimetry confirms that these variants are folded. Data represent mean ± SD of at least two independent experiments. Data are plotted throughout for wild-type CHK2 (full length) in black, wild-type CHK2 (210-531) in pink, and mutants are color-coded: K245A in purple; K255A, light blue; K373A, dark blue; and K465A, brown. K249A (kinase-dead) and K437A exhibited a marked attenuation of basal catalytic activity, and was therefore omitted from further analysis. The melting temperature (T_m) corresponding to the midpoint for the protein unfolding transition is shown for each construct in the table (including 95% Confidence Interval; CI).

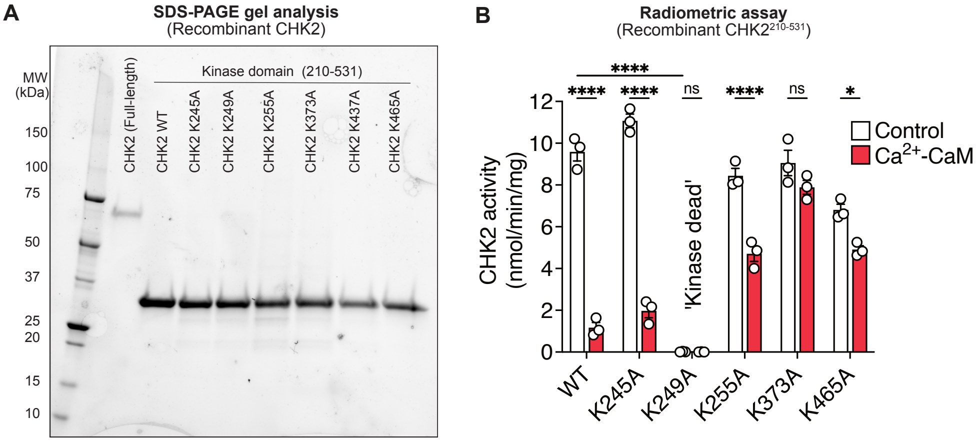

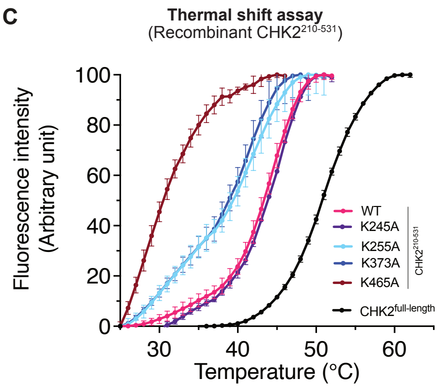

Construct	T_m (°C) [95% CI]
CHK2$^{210\text{-}531}$ WT	43.7 [43.3-44.0]
CHK2$^{210\text{-}531}$ K245A	44.2 [43.9-44.4]
CHK2$^{210\text{-}531}$ K255A	39.8 [38.9-40.7]
CHK2$^{210\text{-}531}$ K373A	39.5 [38.6-40.6]
CHK2$^{210\text{-}531}$ K465A	28.1 [26.7-29.0]
CHK2$^{\text{full-length}}$ WT	50.9 [50.8-51.0]

Supplementary Figure 5 | HDX-MS deuterium uptake plots. Representative CHK2 peptides (black) displaying decreases in deuterium exchange upon binding Calmodulin (CaM; red). Data represent mean ± SD from three independent experiments (n=3). Most error bars are smaller than the size of the point. Source data are provided in Extended Data Excel file.

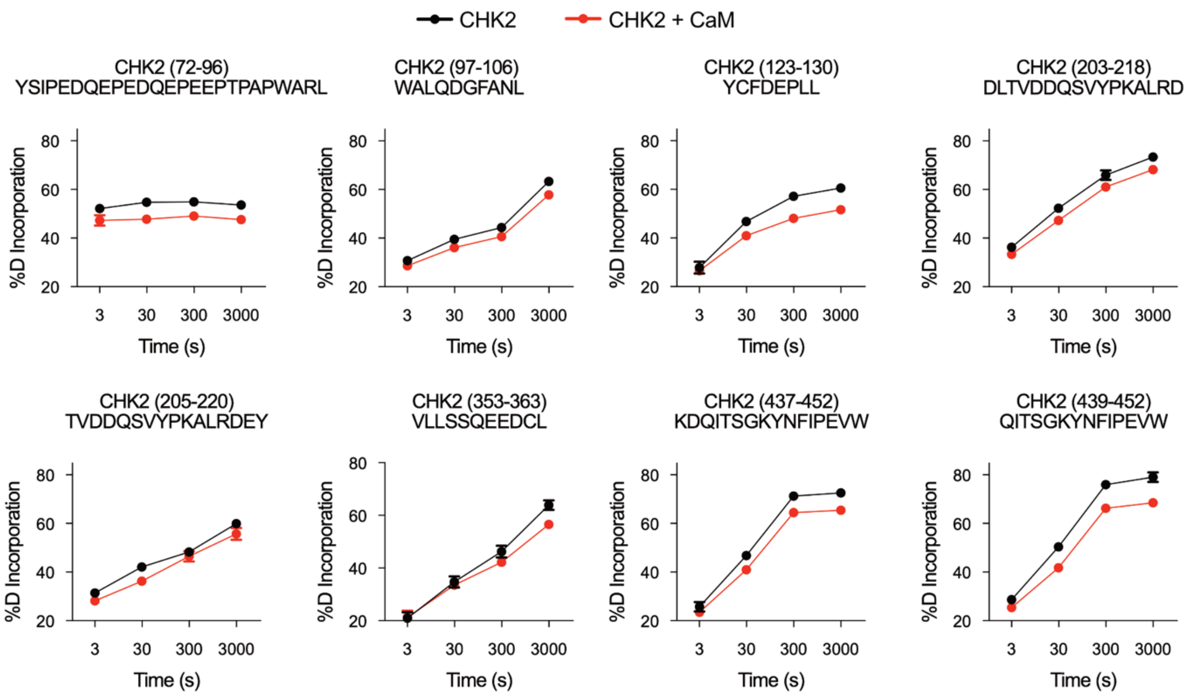

Supplementary Figure 6 | CHK2:Calmodulin AlphaFold PAE plots. Predicted Alignment Error (PAE) of AlphaFold model with CHK2 in complex with Calmodulin. Left hand panel show the per-residue measure of local confidence score (pLDDT) mapped to each Richardson (ribbon) diagram. The rainbow legend for the pLDDT score applies to all models. Right side panel show the PAE plot (in Å).

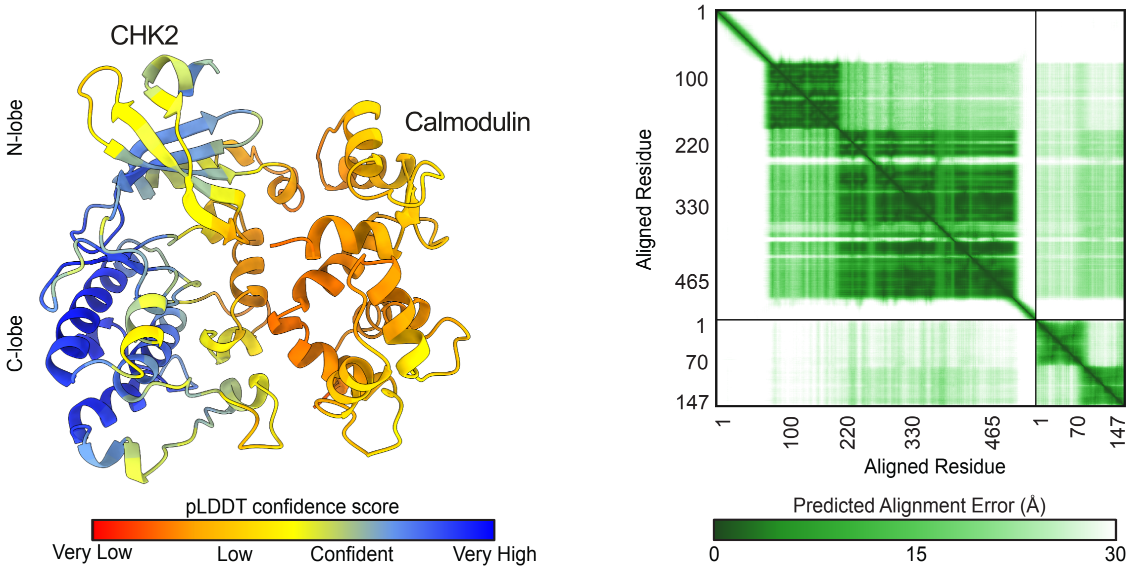

Supplementary Figure 7 | Genotypic validation of CRISPR-edited RPE cells. The endogenous *CHK2* locus of hTERT-immortalized RPE cells was CRISPR edited using the lower donor sequence encoding the K373A mutation (blue) and silent substitutions (green) around the PAM site (yellow). Figure generated using Biorender.

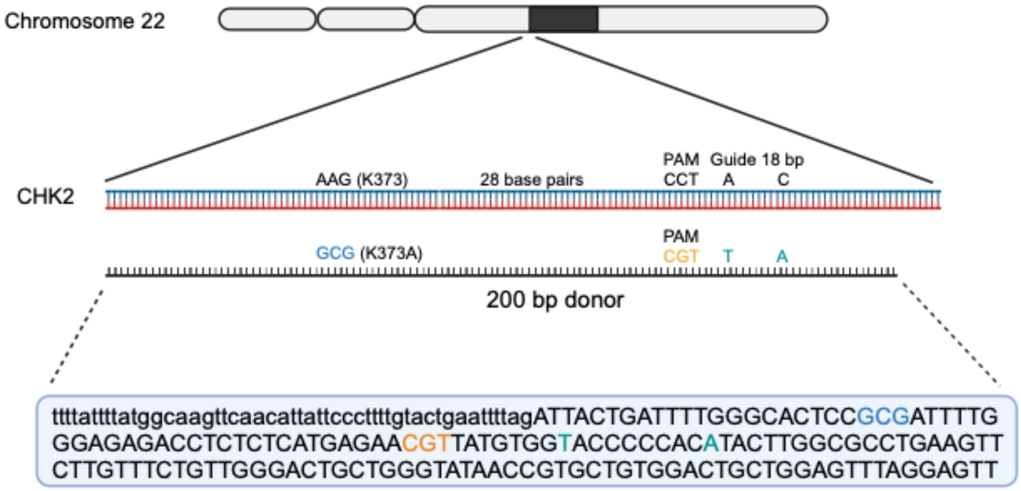

Supplement: online supplementary material 1 [file bcj-482-23-BCJ20253431-s001.pdf]
